# Ketogenic diet therapy for children with super‐refractory status epilepticus in intensive care: International clinical practice recommendations

**DOI:** 10.1002/epi4.70284

**Published:** 2026-05-14

**Authors:** Robyn Blackford, Victoria J. Whiteley, Isobel Hardy, Marisa Armeno, Tessa Bollard, J. Helen Cross, Christin Eltze, Susan George, Charlotte Howard, A. Laura Nijstad, Erin Talzzia, Chris Paget, Samiran Ray, Zoe Simpson, Elizabeth Song‐Pozderac, Agnieszka Szmurlo, Charlene Tan‐Smith, Lisa Vanatta, Elles van der Louw, Natasha E. Schoeler, the Ketogenic Dietitians Research Network

**Affiliations:** ^1^ Ann & Robert H. Lurie Children's Hospital of Chicago Chicago Illinois USA; ^2^ Royal Manchester Children's Hospital Manchester UK; ^3^ Great Ormond Street Hospital for Children London UK; ^4^ Metabolic Research Center (CINME) Buenos Aires Argentina; ^5^ Sydney Children's Hospital Randwick Australia; ^6^ UCL Great Ormond Street Institute of Child Health London UK; ^7^ Sheffield Children's Hospital Sheffield UK; ^8^ Birmingham Children's Hospital Birmingham UK; ^9^ Erasmus University Medical Center Rotterdam The Netherlands; ^10^ Dayton Children's Hospital Dayton Ohio USA; ^11^ Akron Children's Hospital Akron Ohio USA; ^12^ Christchurch Hospital Christchurch New Zealand; ^13^ Phoenix Children's Hospital Phoenix Arizona USA

**Keywords:** epilepsy, high‐fat low carb, ketogenic diet, multidisciplinary, status epilepticus

## Abstract

**Objective:**

We aimed to create practical recommendations to support healthcare teams starting ketogenic diet therapy (KDT) for children with super‐refractory status epilepticus in intensive care settings.

**Methods:**

A literature review was conducted to extract published data on patient selection, diet prescription, diet initiation, monitoring, fine‐tuning, and discontinuation of KDT for super‐refractory status epilepticus. Statements were formulated within each subtopic, and an online survey was distributed to gauge the extent of international agreement with these statements to inform consensus‐based recommendations for initiation and maintenance of KDT in pediatric intensive care settings, with a focus on enteral nutrition. Consensus on a statement for inclusion in the core recommendations was reached if ≥75% of respondents “Agreed” or “Strongly Agreed”. Recommendations from relevant published guidelines or studies were also included.

**Results:**

Twenty‐two relevant manuscripts were identified, and 25 statements were formulated. Seventy‐two healthcare professionals responded to the survey, including dietitians, medics, nurses, intensivists, and a neurophysiologist. Clinical recommendations were made across the following areas, using evidence from published literature, survey agreement, or the clinical expertise of the authors: patient selection and timing of start, preparation for treatment, dietary prescription, diet initiation, monitoring adverse effects, fine tuning, and weaning of KDT. Thirty statements met the criteria for core recommendations.

**Significance:**

These are the first international multidisciplinary recommendations for use of KDT for children with super‐refractory status epilepticus, intended as a sensical guide for dietitians, neurologists, intensivists, and associated healthcare professionals. The majority of the recommendations are based on survey agreement due to a paucity of published evidence.

**Plain Language Summary:**

Children with very severe seizures that do not stop despite medication often need care in intensive care units. This study brings together published evidence and international expert consensus to provide clear guidance on when and how a high‐fat, low‐carbohydrate ketogenic diet can be started safely in this setting. The recommendations highlight early diet initiation, close monitoring, and strong teamwork between doctors, dietitians, nurses, and pharmacists while recognizing that more high‐quality research is still needed.

**Key points**

- These are the first international consensus‐based recommendations for KDT in pediatric SRSE.
- Early KDT initiation in the ICU is recommended, with a rapid start under close monitoring despite limited evidence.
- Medications should be reviewed by a pharmacist, as many should be used with caution alongside a KDT and carbohydrate content should be noted;
- Classical ketogenic diets are most commonly used, but medium‐chain triglycerides can be incorporated;
- The roles of each member of the multidisciplinary team should be agreed; all are essential for preparation, initiation, monitoring, and diet discontinuation.

## 1 INTRODUCTION

Ketogenic diet therapy (KDT) is a group of high‐fat, low‐carbohydrate diets used as an effective treatment option for drug‐resistant epilepsy.^1^ Over the past decade, reports of KDT use in acute situations have increased, particularly for super‐refractory status epilepticus (SRSE), defined as status epilepticus (SE) that persists for at least 24 h after the initiation of general anesthesia, or that recurs following anesthesia reduction or withdrawal.^2^

KDT is described as “particularly useful” for SRSE in international KDT clinical management recommendations, with reported response rates exceeding 70%, compared with an average response rate of approximately 50% when response is defined as >50% seizure reduction.^3^ International consensus recommendations for management of new‐onset refractory status epilepticus (NORSE), including febrile infection‐related epilepsy syndrome (FIRES), suggest that KDT may be considered within 7 days of seizure onset in cases of unknown etiology.^4^ Systematic reviews have reported KDT‐associated cessation of (SR)SE in approximately 60%–70% of children.^5^, ^6^ Although these data are largely derived from uncontrolled studies, case series, and case reports, the findings are nonetheless encouraging.

In recent years, KDT has been increasingly used in pediatric intensive care units as an adjunctive treatment for SRSE, particularly where conventional anti‐seizure and anesthetic therapies have failed or cannot be safely weaned.^7^ While international epilepsy and neurocritical care guidelines acknowledge a role for KDT in these settings, detailed practical guidance on diet initiation, prescription, monitoring, and discontinuation within the constraints of critical care remains limited.^4^, ^8^ Consequently, current practice is variable and often reliant on local expertise and clinical judgment. The recommendations presented here are intended to complement existing intensive care and epilepsy management pathways by providing structured, pragmatic guidance on the safe and timely use of KDT in pediatric intensive care.

The aim of this work was to develop practical, multidisciplinary recommendations to support healthcare teams when initiating—or considering initiation of—KDT for children and young people in intensive care settings. These recommendations are intended to be used alongside specialist input from an experienced ketogenic diet team, with close collaboration to support dietary initiation, monitoring, and ongoing management.

## 2 METHODS

### 2.1 Study group

Invitations for involvement from healthcare professionals currently working with KDT in emergency settings were distributed via the Ketogenic Dietitians Research Network (KDRN), UK Ketogenic professional advisory group (KetoPAG), the international ketogenic dietitian .listserv and British Paediatric Neurology Association (BPNA). Recipients were encouraged to share the invitation with their pediatric neurology and intensive care clinicians. All volunteers had at least 5 years clinical experience in KDT or intensive care settings and were accepted for involvement in the project.

### 2.2 Literature review

A PubMed literature search was performed using the keywords (“ketogenic diet”) AND (“intensive care” OR “critical care” OR “febrile infection‐related epilepsy syndrome” OR “FIRES” OR “new‐onset refractory status epilepticus” OR “NORSE”). No date limitations were set, and only human studies were included.

Data regarding patient selection, diet prescription, diet initiation, monitoring, fine‐tuning, and discontinuation of KDT were extracted from relevant manuscripts.

The Effective Public Health Practice Project (EPHPP) Quality Assessment Tool for Quantitative Studies (https://merst.ca/ephpp) was used to assess the quality of included quantitative studies. The EPHPP includes questions on selection bias, study design, confounders, blinding, data collection methods, and withdrawals and dropouts. Each section is rated as “strong,” “moderate,” or “weak”; collectively, these are used to derive a global rating of “strong” (no weak ratings), “moderate” (one weak rating), or “weak” (two or more weak ratings).

The Appraisal of Guidelines for Research and Evaluation (AGREE II) Instrument^9^ was used to assess the quality of clinical practice guidelines or consensus statements. The tool covers six domains: scope and purpose, stakeholder involvement, rigor of development, clarity of presentation, applicability, and editorial independence. A quality score is calculated for each domain by summing up the scores of the individual items within the domain and scaling the total as a percentage of the maximum possible score for that domain. Domain scores below 40% were classed as “lower quality”, 40%–70% as “moderate quality”, >70% as “higher quality”: thresholds that have been used elsewhere.^10^

“A MeaSurement Tool to Assess systematic Reviews”, AMSTAR 2,^11^ was used to assess the quality of systematic reviews. For narrative reviews, AMSTAR 2 was applied as the closest available structured appraisal framework to allow consistent assessment across included evidence, recognizing that it was not specifically designed for non‐systematic reviews. The tool includes 16 questions, of which 7 are classed as “critical”. Overall confidence in the review is classed as “high” (zero or one non‐critical weakness), “moderate” (more than one non‐critical weakness), “low” (one critical flaw with or without non‐critical weaknesses'), or “critically low” (more than one critical flaw with or without non‐critical weaknesses). An answer of “No” to any of the “critical” questions was classed as a critical flaw, an answer of “No meta‐analysis conducted” (applicable for questions 11 and 15) or a “Partial Yes” were classed as critical weaknesses.

Quality assessment was conducted independently by two of three reviewers (RB, VJW, NES); disagreements were discussed and, if required, the third reviewer was brought in to reach a consensus.

### 2.3 Survey

Key points identified from the literature regarding patient selection, diet prescription, diet initiation, monitoring, fine‐tuning, and discontinuation of KDT for children (ages 0–18 years) in intensive care settings were entered into an online survey (Supporting Information). The aim of the survey was to gauge the extent of international agreement with these specific statements, using a 5‐point Likert scale (Strongly Agree, Agree, Neither Agree nor Disagree, Disagree, Strongly Disagree), to inform consensus‐based recommendations for initiation and maintenance of KDT in pediatric intensive care settings. The focus was on enteral nutrition, as recommendations for ketogenic parenteral nutrition already exist.^12^ The survey was circulated via KDRN, KetoPAG, .listserv and BPNA.

The consensus threshold for inclusion of statements in the core recommendations was ≥75% “Agree” or “Strongly Agree”. Statements that met a majority (>50% “Agree” or “Strongly Agree”) but did not meet the consensus threshold were included in the main body of text (but not the core recommendations) to reflect the variability in clinical practice. Statements that did not meet a majority agreement were reflected in the main body of text but were not included as recommendations. Statements from published consensus guidelines or studies identified in the literature review were also included in the core recommendations if not covered in the survey.

## 3 RESULTS

### 3.1 Literature review

Of 114 manuscripts identified, 22 contained relevant information on KDT (any part in the patient pathway), or relevant information such as glucose and ketone levels in pediatric intensive care settings (Table S1). Twenty‐five statements were produced based on this published evidence and on current clinical practice at the institutions of the study group.

### 3.2 Quality assessment

All quantitative studies, appraised using the EPHPP tool, were assigned a final global rating based on a compilation of scored components (Table S2). All except for the single randomized controlled trial (RCT) scored an overall “weak” rating, most commonly due to confounders and data collection methods. The RCT^13^ received a “strong” rating for all component categories, except for blinding, which was rated “moderate”. Outcome assessors were not blinded to the intervention or exposed status of participants in any of the included studies. The single prospective study included^14^ was classed as “moderate” for selection bias, study design, and blinding components, and as “weak” for withdrawals and drop‐outs. All studies were single‐arm or did not mention differences between groups, so ratings for confounders were “weak”. Data collection relied predominantly on medical record review, with no evidence of validation or reliability testing for tools used. Only the RCT^13^ received a “strong” rating for withdrawals and drop‐outs.

All three literature reviews,^5^, ^15^, ^16^ appraised using AMSTAR 2,^11^ demonstrated critical methodological weaknesses, resulting in an overall rating of “critically low” confidence (Table S3). None of the reviews met key critical domains, with each failing to register a protocol, provide an adequate search strategy, justify study exclusions, conduct a robust risk‐of‐bias assessment, or account for bias when interpreting findings.

Included clinical guidelines, evaluated using AGREE II,^9^ demonstrated substantial variability across domains and between guidelines (Table S4). Honarmand 2024,^17^ Van der Louw 2016,^18^ and Wickstrom 2022^4^ consistently achieved strong ratings across most domains. Others, such as Gomes 2018^19^ and Berger 2019,^20^ obtained lower scores, particularly in Stakeholder Involvement, Rigor of Development, and Applicability. Domain 1 (Scope and Purpose) and Domain 4 (Clarity of Presentation) generally received the highest ratings across guidelines, whereas Domain 5 (Applicability) was the weakest area.

### 3.3 Survey

Seventy‐two responses were received, including 46 dietitians, 19 medical doctors, 3 nurses, 3 intensivists, and 1 neurophysiologist. Most (39/72, 54%) respondents were from the UK, followed by Australia (11/72, 15%), USA (8/72, 11%), Ireland (2/72, 3%), New Zealand (2/72, 3%), Canada (1/72, 1%), Brazil (1/72, 1%), and India (1/72, 1%); 7/72 (10%) were unknown (the country of place of work was not explicitly requested, but most were evident from the registered professional body information provided). Of the 72 respondents, 28 (82%) had worked with patients in the intensive care unit with super‐refractory status epilepticus for 0–5 years; 20 (28%) for 5–10 years and 24 (23%) for >10 years.

## 4 RECOMMENDATIONS

### 4.1 Patient selection and timing of start

#### 4.1.1 Recommendations 1.1 and 1.2

International guidelines for management of NORSE including FIRES^4^ support use of KDT within the first week of onset with inadequate response to first‐line therapies. KDT may also serve as an alternative therapeutic option in SRSE (including non‐NORSE) where antiseizure medications (ASMs) used to manage status epilepticus are unable to be weaned.^19^

In the survey, there was consensus that existing guidelines regarding medical management of NORSE and FIRES should be followed for patient selection and timing of KDT initiation.^3^, ^4^, ^19^

### 4.2 Preparation for treatment

#### 4.2.1 Recommendations 2.1–2.2

Best practice for most patients with epilepsy starting KDT is to await free carnitine, plasma amino acids and urine organic acid profile results to rule out disorders of fatty acid transport or oxidation.^3^ However, this is likely to cause a delay to starting dietary treatment and so centers may wish to request these investigations as soon as possible after admission with SE, if first‐line treatments are unsuccessful. For intensive care admissions with SE, where outcomes are heavily dependent on the time lapse to controlling seizures, KDT may be introduced before these results are available, as metabolic activity can be closely monitored. There was majority (58%) agreement but no consensus for starting KDT without these screening results available, as long as there were no pre‐existing risk factors for fatty acid oxidation disorders, such as unexplained hypoglycemia, unexplained metabolic acidosis, deranged liver enzymes, and elevated creatine kinase. If there is a concern about a possible metabolic etiology, complete the appropriate screening investigations or seek advice from the metabolic team prior to initiating KDT.

The pharmacist plays an important role in reviewing medications to assess for potential interactions with dietary treatment and carbohydrate, sugar alcohol, and alcohol content. Enteral medications should be switched to the lowest carbohydrate formulations available. The pharmacist may choose to make a pragmatic decision regarding the most suitable formulation, considering the safety and feasibility of administering solid‐dosage forms via feeding tubes, which may be outside manufacturer's product licenses. Medication review or change should not delay KDT initiation.

Caution should be taken when considering KDT alongside propofol, due to the increased risk of propofol‐related infusion syndrome (PRIS). A fatal case of PRIS was reported in a 10‐year‐old boy when KDT was initiated,^21^ although a retrospective analysis of 165 “anesthetic encounters” with propofol infusions in patients on KDT identified no PRIS cases.^22^ In practice, it may not be feasible to use alternative anesthetic agents, and the individual risk–benefit ratio should be cautiously considered, with appropriate monitoring for PRIS. If KDT is to be considered alongside propofol, then the fat content from propofol (0.1 g/mL of solution) should be included in the dietary prescription.

A potential barrier to starting KDT as soon as possible may be the need for inotropic or vasopressor support. In these cases, inotropes reduce blood flow to the gut, increasing the risk of necrotizing enterocolitis. Due to the urgent nature of the situation, it may be worth discussing with the medical team while these interventions are being used to fully assess the risks and benefits of KDT initiation at that time.

There are other medications that should be used with caution while on KDT, requiring specific additional monitoring.^23^, ^24^ Concomitant usage of corticosteroids increases blood glucose, which can cause initial hyperglycemia and a subsequent decrease of ketogenesis, although these are commonly prescribed for short periods. Intravenous and oral carbonic anhydrase inhibitor medications (i.e. topiramate, zonisamide, and acetazolamide) may cause metabolic acidosis, requiring the use of a bicarbonate or citrate supplementation for correction. The effects of pentobarbital/pentobarbitone on glucose metabolism may present a challenge to achieving ketosis. Medical teams should also note that valproic acid levels have been shown to decrease^24^ and phenobarbital levels increase^25^ in people on KDT.

Intravenous medications should also be reviewed, with specific attention to diluents, as they may need to be switched to sodium chloride where compatible (^12^ gives recommendations on ketogenic parenteral nutrition).

See Table 1 for a summary of points regarding pre‐diet preparation for KDT.

**TABLE 1:** Ketogenic dietary therapy management in pediatric intensive care.

Phase	Category	Recommendations
Pre‐diet preparation	Baseline Assessment	Obtain baseline labs including carnitine profile, urine organic acids, full blood count with platelets, electrolytes (including serum bicarbonate, total protein, and calcium), liver function tests, renal profile, and lipid profile
	Screening Considerations	Await carnitine and urine organic acid results if fatty acid oxidation disorder is suspected; if no such risk factors are present, consider initiating KDT before results are available
	Medication Review	If on propofol and alternative agents are not possible, cautiously consider the risk–benefit ratio before starting KDT; change medications to the lowest carbohydrate form
	Team Responsibilities	KDT should be planned and monitored by an experienced ketogenic dietitian; determine appropriate timing for initiation
Diet prescription	Energy	Follow critical care international guideline calculations for age and weight
	Protein	Meet estimated requirements for weight and age if able; meet safe low intake requirements as a minimum; prioritize protein over carbohydrate
	Carbohydrate	Gluconeogenesis may be sufficient to maintain normoglycemia with minimal carbohydrate intake, but glucose levels must be monitored
	Ketogenic Ratio	No specific initial ratio is recommended, but 3:1 and 4:1 are most commonly used
	Fat	If protein needs cannot be met within the target ratio, replace some long‐chain fat with MCT to optimize ketosis
	Fluids	Tailor to the patient's clinical status in collaboration with the intensive care team; use the lowest glucose amount possible in IV fluids to maintain target ketosis and normoglycemia
	Micronutrients	Ensure 100% reference intake for vitamins, minerals, and electrolytes unless significant disturbances are present
Monitoring and adjustment	Glucose	Monitor at least every 4–6 h during diet initiation; treat hypoglycemia if glucose falls below age‐specific thresholds
	Ketones	Stable ketosis is defined as beta‐hydroxybutyrate >2 mmol/L for 48 h
	Laboratory Monitoring	Monitor biochemical labs and clinical side effects; additional labs may include full blood count with platelets, electrolytes, liver function tests, renal profile, lipid profile, free and total carnitine, and selenium
	Daily Review	Review diet prescription daily until stable ketosis is achieved
	Adjustment	Adjust the ketogenic ratio and/or percentage of MCT fat to maintain at least minimum protein intake and glucose levels
	Duration	Continue KDT for a minimum of 2 weeks once stable ketosis is achieved
	Reassessment	If efficacy is unclear after 2 weeks, consider continuing KDT for an additional 2 weeks
Discontinuation	Short‐Term Use	If KDT has been used for <4 weeks, or >4 weeks with adverse side effects, wean over 12–48 h
	Longer‐Term Use	If KDT has been used for >4 weeks, wean over 3–5 days

### 4.3 Dietary prescription

#### 4.3.1 Recommendations 3.1–3.13

The ketogenic prescription should be calculated by a ketogenic dietitian or, at least, with support from a dietitian experienced in intensive care and/or KDT.

##### Type of ketogenic diet therapy

No consensus was reached regarding which ratio to use when commencing KDT, nor at what stage to consider use of medium‐chain triglycerides (MCT). The initial diet prescription must consider the following factors:

- Clinical status
- Nutritional status
- Risk of hypoglycemia (higher in infants)
- Optimal protein provision
- Tolerance of MCT

Starting ratios of 3:1 or 4:1 have been reported in the literature: a 4:1 ratio was used in 10 patients, with ketonuria reached within 2–4 days^26^; a 4:1 starting ratio was used in a case report^27^; a 3:1 starting ratio was used in children <18 months and a 4:1 ratio in children >18 months old (*n* = 7).^28^ There was majority agreement (69%) but no survey consensus that diet ratios higher than 4:1 can be considered to compensate for carbohydrates in medications, as has been reported in the literature.^14^

See Table S5 for a list of commonly used prescribable products available to support medical KDT. The availability and formulation of these products may vary between countries and healthcare systems.

Blended KDT may be used for enterally fed patients, but no reports were identified from the literature, possibly due to food safety concerns or following USA clinical consensus guidelines that fiber should not routinely be used in critically ill patients^29^ (and most blended diets are high in fiber). Additionally, critically ill patients are more likely to be fed continuously rather than by bolus, meaning that it is not practically possible to deliver blended diets, which tend to be given via syringe bolus and used within 2 h when kept at room temperature.^30^, ^31^ As such, healthcare teams may wish to explore modular or formula‐based options as first‐line.

##### Energy requirements

Measuring resting energy expenditure via indirect calorimetry is the gold standard to prevent over‐ or underfeeding, and to allow for measurement of the nutrient utilization coefficient during KDT,^32^ but it may not always be possible to do.^33^ A detailed nutritional history and growth status of the patient should be completed to better understand baseline caloric needs.

Estimated energy requirements can be calculated using intensive care guidelines (ESPNIC,^34^ ASPEN & SCCM,^35^ or ESPEN^36^), taking into account type and severity of illness, degree of malnutrition, current medications, baseline physical ability, ventilation status, seizures and/or hypertonia.

Using Resting Energy Expenditure for age when calculating goal caloric intake is important due to sedation and risk of overfeeding. Hypocaloric feeds may be necessary at the start of KDT, but gradually increased to the patient's potential goal as they recover from SE and sedation is decreased.

##### Protein and carbohydrates

International guidelines for intensive care (ESPNIC,^34^ ASPEN & SCCM,^35^ or ESPEN^36^) should be aimed for, including progressive increases in protein provision. If these levels cannot be met within the KDT prescription, it should be determined, on a case‐by‐case basis and with caution, whether the level of ketosis takes priority over meeting protein needs (or vice versa) in the short term. Safe low protein intakes (Joint WHO/FAO/UNU Expert Consultation^37^) should be met as a minimum. In many cases, protein provision on KDT can be optimized over provision of carbohydrates while maintaining or increasing the ketogenic ratio, with endogenous glucose production by gluconeogenesis and glycogenolysis sufficient to maintain normoglycemia. However, in critical illness, endogenous glucose production is disrupted^16^ and so blood glucose levels must be monitored closely when transitioning carbohydrates in feeds to protein.

##### Fat

The classical ketogenic diet (KD) is traditionally calculated using only long‐chain triglycerides (LCT). However, it is now accepted practice to replace some LCT with MCT to help promote ketosis, and also improve feed tolerance due to the reduced pressure on the liver for MCT metabolism. This may be particularly helpful to counteract ketosis‐lowering effects from specific medications, or if carbohydrate from medication is unavoidable. If calories from MCT are calculated as 9 kcal/g (aligning with calorie calculations in commercial feeds), the dietitian may wish to document the calorie deficit from calculating the calorie content of MCT as 8.3 kcal/g^38^ and monitor growth accordingly.

Arayakarnkul and Chomtho^39^ used MCT at 45%–55% of total energy (*n* = 8) in the initial KD prescription; no gastrointestinal side effects were reported in this patient group, but teams should be aware that MCT can cause gastrointestinal upset, such as diarrhea and vomiting.

In diets with higher percentage intakes of MCT or feeds made up of individual macronutrient modules, it is important to ensure adequate essential fatty acid (EFA) intake.^40^ Recommendations from the UK Scientific Advisory Committee on Nutrition (SACN) state that linoleic acid (*n* − 6) should provide at least 1% of total daily energy and alpha‐linolenic acid (*n* − 3) should provide at least 0.2%.^41^ If supplementation is required, also consider intake from products such as Calogen™, walnut or flaxseed oil (high sources of omega 3 fatty acids); walnut oil typically provides 55 g linoleic acid and 12 g alpha‐linolenic acid per 100 mL. If EFAs are supplemented, levels of vitamins A and E should be monitored regularly due to the high levels of fat‐soluble vitamins in EFA oils.

##### Fluid

Fluid management will be led by the intensive care medical team and must be tailored to the patient's clinical status, organ function, age and body weight, fluid balance, serum electrolytes, acid–base status, and hemodynamic status. The feed allowance is calculated once deductions have been made for fluid restriction, intravenous or enteral medications and flushes. Clear communication with the medical team regarding regular review of fluid allowance is essential to enable adequate feed provision.

##### Vitamins and minerals

There was majority agreement (53%) but no survey consensus that micronutrient requirements should meet at least 80% of reference nutrient intakes from day 1 of treatment. The target plan should meet full requirements for vitamins, minerals, and electrolytes. Further supplementation of vitamins and minerals can be considered if the target feed delivery is not reached within 2–3 days. It is generally accepted in practice that, on initiation, the plan might not be nutritionally complete, but this will need to be reviewed regularly, especially given the risk that the feed target may not be reached.

See Table 1 for a summary of points regarding diet prescription.

### 4.4 Diet initiation

#### 4.4.1 Recommendations 4.1 and 4.2

There was majority agreement (68%) but no consensus for weaning onto KDT within 12–48 h. Four survey respondents commented that their practice is to start at the target plan immediately, with no “weaning on” period in terms of calories or ketogenic ratio, supported by the close biochemical and clinical monitoring possible in the intensive care setting, and the urgency to control seizures. The maximum reported length of time to wean onto “full” KDT was 5–7 days. A more gradual introduction may reduce the risk of hypoglycemia and gastrointestinal side effects but may delay seizure control.

No consensus was reached regarding the way to advance KDT. 82% respondents selected “Starting at target calorie delivery, advancing by 1 ratio point per day to reach target ratio by day 3”, “Starting at the target ratio, advancing by 1/3 target calories per day to reach target calorie delivery by day 3”, *or* “Either” of these options. Survey comments (9/31) highlighted that the initial calorie provision and calorie advancement plan should be guided by international intensive care guidelines (ESPNIC,^34^ ASPEN & SCCM,^35^ or ESPEN^36^) or usual intensive care feeding algorithms. In 11 of 31 comments on this question, practitioners recommended starting at the target ketogenic ratio, whereas 8 would progress the ratio in a stepwise fashion. There was majority agreement (68%) but no consensus that KDT should be introduced over a period of 12–48 h and that ketones should be monitored 4–6 hourly while weaning onto KDT (63% agreement).

Caraballo et al. (2014) fasted 10 patients for 24 h prior to KDT initiation, of which one had severe vomiting and hypoglycemia.^26^

There was majority agreement (63%) but no consensus that international intensive care guidelines (ESPNIC,^34^ ASPEN & SCCM,^35^ or ESPEN^36^) should be followed regarding route and progression of feeding. Individual risk of refeeding syndrome should also be considered.

Table 2 outlines an example of how to initiate KDT in this setting.

**TABLE 2:** Example initiation of ketogenic dietary therapy in pediatric intensive care unit.

*Patient details*: 10‐year‐old child, previously fit and well, appropriate growth history, no dietary restrictions. Admission to pediatric intensive care unit in super‐refractory status epilepticus. Normal intake estimated at 2000 kcal daily at 36.2 kg and 141.9 cm *Target classical ketogenic diet prescription: 3:1 ratio* Estimated requirements whilst ventilated and sedated and apyrexial: 1489 kcal (Schofield), 42.1 g protein (RNI), 1277 mL fluid (70% of normal fluid requirements due to being intubated and ventilated) *Goal macronutrients: 144 g fat, 42.1 g protein and 6 g carbohydrate*

	Fat (g)	Protein (g)	Carbohydrate (g)
Day 1			
2:1 classical ratio (Target: 135 g fat, 42.1 g protein, 25.6 g carbohydrate)			
913 mL 1.5 kcal/mL 4:1 ketogenic formula	135	28.2	5.6
15 g protein powder	‐	13	‐
20 g carbohydrate polymer	‐	‐	19.2
Additional water to target volume of 1000 mL	‐	‐	‐
Total	135	41.2	24.8
Day 2			
3:1 classical ratio (Target: 144 g fat, 42.1 g protein, 6 g carbohydrate)			
973 mL 1.5 kcal/mL 4:1 ketogenic formula	144	30	5.9
13 g protein powder	‐	11.3	‐
Additional water to target volume of 1000 mL	‐	‐	‐
Total	144	41.3	5.9

*Note*: To feed continuously 42 mL/h to provide 1000 mL in 24 h. Additional fluids to be provided by medications and IV fluids, avoiding glucose.

Further examples are included in Tables S6 (a 4 year old) and S7 (infant, including addition of MCT).

### 4.5 Monitoring and adverse effects

#### 4.5.1 Recommendations 5.1–5.8

##### Monitoring

Biochemical monitoring may be required more frequently than for a non‐emergency start, depending on the speed of introduction of KDT. ESPEN guidelines^36^ may guide the frequency of biochemical monitoring (with recognition that not all parameters are applicable or appropriate for each individual):

- Blood glucose should be measured initially (after ICU admission or artificial nutrition initiation) and at least every 4 h for the first 2 days.
- Electrolytes (potassium, magnesium, phosphate) should be measured at least once daily for the first week.
- In patients with refeeding hypophosphatemia (<0.65 mmol/L or a drop of >0.16 mmol/L), electrolytes should be measured 2–3 times/day and supplemented if needed.
- In patients with refeeding hypophosphatemia, energy supply should be restricted for 48 h and then gradually increased.

In addition, full blood count with platelets, serum bicarbonate, total protein and calcium levels, liver function tests, renal and lipid profiles, and free and total carnitine may be monitored in accordance with international best practice recommendations for KDT^3^ (vitamin D and selenium levels are included in these recommendations, but usually would not be monitored frequently). Fasting lipid profiles may be conducted where further monitoring is required, for example, for hypertriglyceridemia, but local teams should weigh up the advantages and disadvantages of short‐term lipid profile “abnormalities” against potential or actual seizure improvement. Recommendations for biochemical monitoring have been published for children on KDT parenteral nutrition, but there was little consistency in what tests were conducted, nor the frequency of monitoring from the authors' international survey of clinical practice.^12^

Monitoring for metabolic acidosis, lipemia, rhabdomyolysis, and myocardial failure is pertinent to avoid PRIS if KDT is used alongside propofol. In these cases, triglycerides should be monitored 12 hourly due to the increased risk of hypertriglyceridemia and resultant pancreatitis.^42^

A medication review should be undertaken with the multidisciplinary team, particularly if there are challenges with achieving or maintaining ketosis (for example, with concomitant usage of corticosteroids, pentobarbital/pentobarbitone, or phenobarbital), increased risk of metabolic acidosis (for example, carbonic anhydrase inhibitors), or continuing seizures (consider efficacy of specific ASMs alongside KDT). Strategies to mitigate potential interactions include careful monitoring of carbohydrate intake and consideration of alternative anesthetic agents when feasible. Increasing the diet ratio or adding MCT may help to counteract the ketosis‐lowering effects of some medications.

Both diuretics and KDT independently promote the excretion of electrolytes. Combining diuretic medications with KDT can exacerbate those electrolyte imbalances, potentially leading to adverse effects such as dehydration and hypokalemia.^43^ To avoid potentially dangerous side effects, supplementation with electrolytes, particularly sodium, potassium, and magnesium while using diuretics, and maintaining adequate fluid intake to support kidney function and electrolyte balance, is common clinical practice.

See Table 1 for a summary of diet monitoring and adjustments.

##### Hypoglycemia

The survey did not ask about the “threshold” at which to treat hypoglycemia, although guidance on this was requested by respondents in the comments section. There are discrepancies in the definition of hypoglycemia amongst centers, translating to differences in the acceptable lower limit for glucose before treatment. In a systematic review of hypoglycemia in critically ill children,^15^ authors defined hypoglycemia as <40–45 mg/dL (<2.2–2.5 mmol/L) in neonates and <60–65 mg/dL (<3.3–3.6 mmol/L) in children, although it should be noted that individuals on KDT typically have lower blood glucose levels than individuals not on KDT. Agreement on the accepted lower blood glucose limit, both when the patient is in ketosis or not, should be made with the medical team and communicated to all staff caring for the patient prior to initiation of dietary treatment. Recurrent episodes of hypoglycemia should warrant recalculation of the KD plan. Hypoglycemia is more commonly reported in infants,^44^, ^45^, ^46^, ^47^ with international guidelines recommending a minimum of twice daily glucose monitoring^18^ and more frequent monitoring in neonates, perhaps 4–6 times daily.^46^ Blood glucose monitoring is generally not required once therapeutic ketosis is achieved (ketones >2 mmol/L for 48 h); however, in the intensive care setting, glucose monitoring may be continued on medical advice.

##### Metabolic acidosis

The survey did not specifically ask about current practice regarding metabolic acidosis. One may be guided by recommendations for children on KDT who undergo general anesthesia for surgery: for procedures longer than 3 h, serum pH or bicarbonate levels should be monitored preoperatively and every 2–3 h during the procedure to avoid acidosis and precipitating bicarbonate levels.^48^ If baseline bicarbonate levels are on the lower end of the normal range (approximately 20–28 mmol/L, but varies depending on age and center), monitoring is also advised for shorter procedures. If a patient is in metabolic acidosis, the underlying cause should be investigated and intravenous bicarbonate administered to correct acidosis, according to each center's KDT protocol. Fluid intake should be optimized.

##### Hyperketosis

The survey did not specifically ask about current monitoring practices, but survey respondents commented that they would appreciate guidance on managing (the risk of) excess ketosis. Signs and symptoms of high ketones are nausea/vomiting, lethargy, facial flushing, and Kussmaul breathing (with associated metabolic acidosis), which may be difficult to identify in this patient group. Some patients can tolerate higher levels of ketosis without clinical signs compared to others. Hyperketosis can be defined as ≥5 mmol/L (some centers use 6 mmol/L) and should be treated with 2–4 grams of carbohydrate or 40 milliliters of a 5% glucose‐containing intravenous fluid. This tends to be clinical practice for KDT in non‐emergency settings, as reported in the recently published best practice recommendations for the management of CKD and modified KDs.^49^ Guidelines for infants recommend checking blood ketones twice daily during diet initiation.^18^ During maintenance, monitoring of ketosis should be continued at the advice of the medical team.

##### Constipation

Constipation is common both in individuals on KDT^50^ and in patients on intensive care.^51^ Most commercially available ketogenic feeds contain small amounts of fiber, but this may be insufficient to meet recommended intakes, and many dietary fiber supplements contain some digestible carbohydrates. Medical management with non‐absorbable or low‐carbohydrate bulk‐forming or osmotic laxatives may be preferable to reduce the risk of or to manage constipation. Formulas or feeds containing MCT may also be helpful.

##### Diarrhea

A medical review of the cause of diarrhea should be undertaken prior to any dietary modifications. Diarrhea can be a side effect of MCT and so reducing MCT (if being used), potentially followed by a slower reintroduction, may reduce symptoms. In feeds not containing MCT, reducing the ratio of the feed may be considered, depending on the severity of symptoms. Partially hydrolyzed formulas may also help in patients with malabsorption.

##### Reflux and vomiting

A medical review of the cause of reflux or vomiting should be undertaken prior to any dietary modifications. Anti‐reflux medication, if already used, should be optimized, ensuring appropriate, low carbohydrate or carbohydrate‐free formulations. Vomiting can be a side effect of MCT and so reducing MCT (if being used), potentially followed by a slower reintroduction, may reduce symptoms. In feeds not containing MCT, reducing the ratio of the feed may be considered, depending on the severity of symptoms. There is no evidence to suggest that either continuous or intermittent/bolus feeds are superior in delivering gastric feeds in critically ill children,^34^ but one may consider altering feeding rates and allowing some breaks in the feed to allow for gastric emptying. For those with feeding intolerance, it may be necessary to consider adjustments, especially in the case of large gastric residual volumes. If, despite these adjustments, vomiting is still a concern or there is a risk of aspiration, post‐pyloric feeding can be considered.^34^

### 4.6 Fine tuning

#### 4.6.1 Recommendations 6.1–6.3

The diet prescription may be adjusted as frequently as twice daily, based on medical and metabolic parameters, including seizures and ketosis, to optimize outcomes and response. Efficacy should be assessed by managing intensive care and/or neurology medical teams, in conjunction with dietitians. There is no specified amount of time for which to continue KDT in ICU before determining whether it is effective or not; this decision should be taken on a case‐by‐case basis, taking into account what other treatments have been started or adjusted since the diet was introduced, as these may have impacted dietary effectiveness and/or made effectiveness difficult to determine, together with consistency of ketosis and any potential side effects. In a retrospective cohort study, most responders were seen within 2 weeks, but there were additional responders between 2 weeks and 1 month.^52^ In a systematic review including children and adults with SRSE treated with KDT, the probability of SRSE cessation was 50.5% 7 days after KDT initiation, 33.2% at 14 days, and approximately 25% at 21 and 28 days post KDT initiation.^6^ In a systematic review of children with SRSE treated with KDT, there were cases of response to KDT as late as 19 days after initiation of dietary treatment.^5^

### 4.7 Fine‐tuning key points

- Diet prescription should be reviewed daily until stable ketosis is achieved.
- Adjust the ketogenic ratio and/or percentage of MCT fat to maintain at least minimum protein intake and glucose levels.
- Continue KDT for a minimum of 2 weeks once stable ketosis is achieved.
- If efficacy is unclear after 2 weeks, consider continuing KDT for 2 further weeks.

### 4.8 Weaning

#### 4.8.1 Recommendations 7.1–7.3

There was majority agreement (60%) but no consensus for discontinuing KDT over a 24–48 h period if KDT is not effective. The rate of wean should take into consideration how well the diet has been tolerated and how long it has been followed. For a patient on KDT for less than 4 weeks, or for a patient on KDT for longer than 4 weeks but with side effects that outweigh the need to continue KDT, a rapid wean over 12–48 h may be acceptable. For those on KDT for longer than 4 weeks without seizure improvement, a wean over 3–5 days may be considered. If seizures increase or clinical status deteriorates, one can return to the previous step in the weaning plan—often the most recent ketogenic ratio. If KDT is effective and there is no current plan for weaning, the diet can be continued for at least 6 months after SE resolution. The ideal duration of KDT following SE is unknown but is anecdotally described as approximately 6 months; this seems to refer to the positive outcomes with KDT for those with infantile spasms.^53^

##### KDT discontinuation key points

- For a patient who has been on diet for <4 weeks, or >4 weeks but with adverse side effects, KDT can be weaned over 12–48 h
- For a patient who has been on diet for >4 weeks, KDT can be weaned over 3–5 days

**FIGURE 1:**
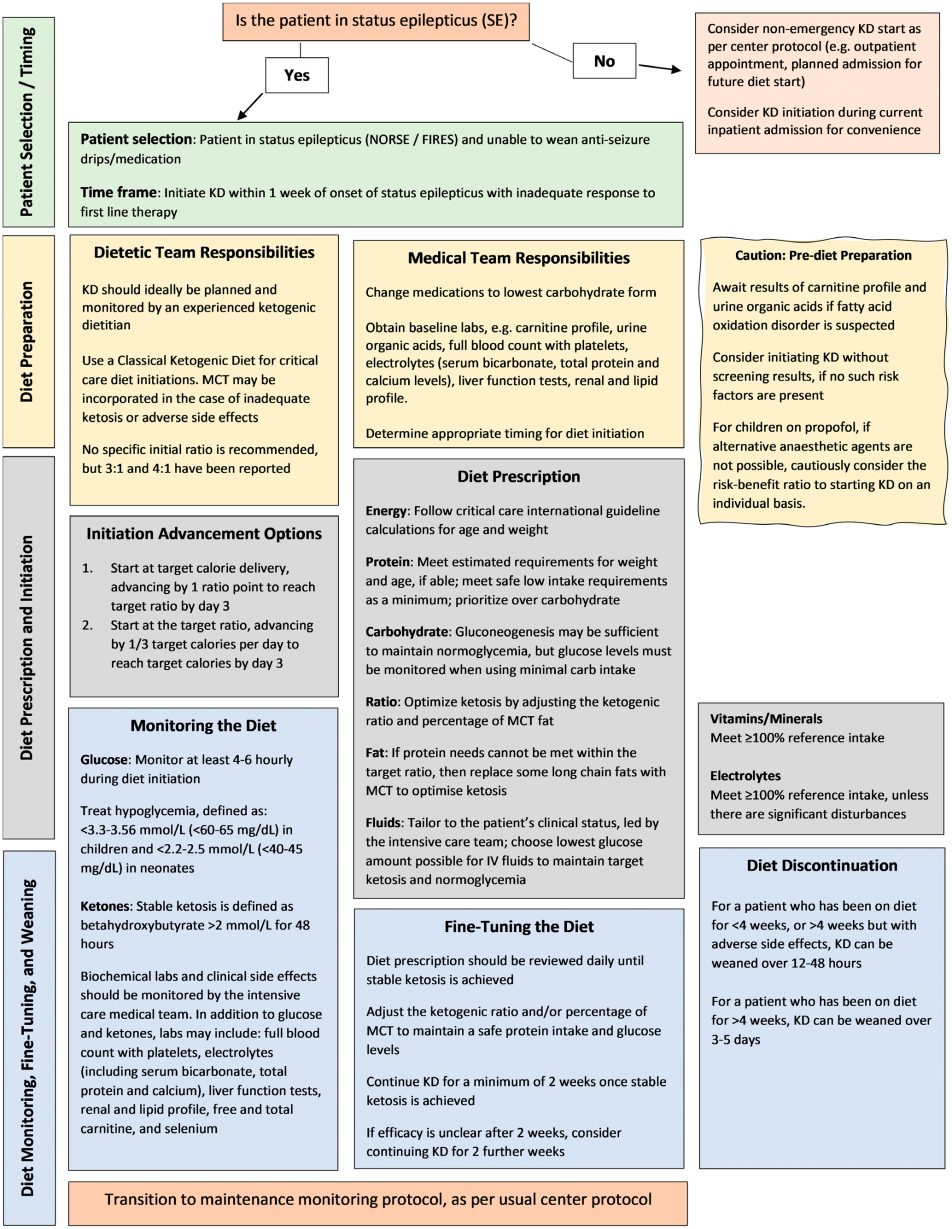
Summary flowchart of recommendations. FIRES, febrile infection‐related epilepsy syndrome; IV, intravenous; MCT, medium‐chain triglyceride; NORSE, new‐onset refractory status epilepticus. These recommendations are for children who are enterally fed; for children who require parenteral nutrition, refer to published clinical practice guide.^12^

See Figure 1 for a graphical summary of recommendations. Table 3 outlines the core recommendations.

**TABLE 3:** Core recommendations.

Recommendation		Grading of recommendation
	*Patient selection and timing of start*	
1.1	Ketogenic dietary therapy should be started within 1 week of new onset refractory status epilepticus (NORSE) as per international consensus recommendations	A1
1.2	Ketogenic dietary therapy should be started within 1 week of super‐refractory status epilepticus (SRSE) where intravenous anti‐seizure medications (ASM) for SRSE management are unable to be weaned in the intensive care unit	A1 and GPP (79%)
	*Preparation for treatment*	
2.1	Where risk factors for disorders of fatty acid oxidation disorders (hypoglycemic episodes, deranged liver enzymes, elevated creatine kinase, history of poor exercise tolerance) are present, await confirmation of normal results of carnitine profile and/or organic acids	GPP (83%)
2.2	Prior to, or during, initiation of ketogenic dietary therapy, medications, supplements and fluids should be reviewed (ideally by a member of the pharmacy team) and either stopped or switched to the lowest carbohydrate‐containing equivalents where possible	GPP (88%)
	*Dietary prescription*	
3.1	A classical ketogenic diet (CKD), with or without the inclusion of medium‐chain triglyceride fats, tends to be the diet type of choice when starting ketogenic dietary therapy in the intensive care setting, or as emergency starts	A2
3.2	International intensive care guidelines (ESPNIC, ASPEN & SCCM, or ESPEN) should be followed when calculating energy requirements	GPP (78%)
3.3	Protein provision should meet at least the safe low intake recommended by the Joint WHO/FAO/UNU Expert Consultation (2007). N.B. This is accepting that the gold standard would be to meet estimated requirements as per international intensive care guidelines (ESPNIC 2020; ASPEN & SCCM 2017; ESPEN 2018)	GPP (77%)
3.4	There is no evidence on which to base a safe lower recommendation for carbohydrate provision. However, blood glucose levels must be monitored closely when negligible carbohydrates are given	A1
3.5	The replacement of some long‐chain triglyceride (LCT) fat with medium‐chain triglyceride (MCT) fat should be considered if protein needs cannot be met at the target ratio	GPP (82%)
3.6	Once minimum protein requirement is met, If MCT fat is being used, the ratio and percentage of MCT should be adjusted to optimize ketosis and minimize adverse effects, while maintaining safe blood glucose levels	A1
3.7	Essential fatty acids should be considered in diets with a large proportion of calories from MCT fat sources, with the aim to achieve Linoleic Acid (*n* − 6) ≥1% of total daily energy and Alpha‐linolenic acid (*n* − 3) ≥0.2% of total energy (SACN)	A2
3.8	The fluid allowance for feeds should be agreed with the intensive care team	GPP (96%)
3.9	If intravenous fluids are required, a carbohydrate‐free, isotonic solution is advised, or an isotonic glucose‐containing fluid at the lowest glucose concentration needed to maintain the ketosis target and normoglycemia (GOSH guidelines, unpublished)	GPP (90%)
3.10	The target dietetic plan should meet ≥ 100% reference nutrient intake for electrolytes, unless there are significant biochemical disturbances which should be accounted for in the feed	A2
	*Initiation*	
4.1	There is no set way to advance ketogenic dietary therapy in this setting, but either of the following advancement plans may be considered (in order of preference): Starting at target calorie delivery, advancing by 1 ratio point per day to reach target ratio by day 3 OR Starting at the target ratio, advancing by 1/3 target calories per day to reach target calorie delivery by day 3	GPP (82%)
	*Monitoring and adverse effects during follow‐up*	
5.1	Blood glucose should be monitored at least 4–6 hourly during diet initiation	GPP (89%)
5.2	Cut‐off levels for treatment of hypoglycemia should be agreed amongst the healthcare team, depending on whether hypoglycemia is with or without ketosis (thresholds for treatment may be lower when the patient is in ketosis)	A2
5.3	Serum ketone levels are the preferred method for monitoring ketones	GPP (92%)
5.4	Therapeutic ketosis can be defined as ketones >2 mmol/l for a period of 48 h	GPP (78%)
5.5	Monitoring of biochemistry and clinical side effects should be led by the intensive care medical team in accordance with Kossoff et al. (2018)	GPP (88%)
5.6	To reduce the risk of constipation, fluid allowance should be optimized where possible in agreement with the intensive care team	GPP (96%)
5.7	In the case of constipation, dietary/supplement fiber intake should be reviewed and optimized where possible, alongside medical management	GPP (82%)
5.8	In the case of diarrhea with no medical cause, a reduction in MCT fat may be considered	GPP (83%)
5.9	In the case of vomiting or reflux, dietary adjustments such as route (including gastric vs post‐pyloric), rate and volume of feed, ketogenic ratio, and feed positioning should be reviewed, alongside medication addition/adjustment, where applicable	GPP (92%)
	*Fine tuning*	
6.1	The dietary prescription should be reviewed daily until ketosis (ketones >2 mmol/l for 48 h) is achieved	GPP (93%)
6.2	The diet should be continued for a minimum of 2 weeks once stable ketosis is achieved	A2 and GPP (86%)
6.3	If efficacy is unclear at 2 weeks, continuing the diet for a further 2 weeks (to a total of 4 weeks) should be considered if not contraindicated	A2 and GPP (82%)
	*Weaning*	
7.1	Consider a rapid wean over 12–48 h if patient has been on diet for <4 weeks; or >4 weeks but with difficult‐to‐manage side effects	A1
7.2	Consider a moderate wean of diet over 3–5 days if patient has been on diet for >4 weeks	A1
7.3	If the diet has been effective for resolution of SE, then KDT may be followed for approximately 6 months prior to considering discontinuing diet	A1

*Note*: Grades: A1—Evidence‐based (published consensus guidelines). A2—Evidence‐based (literature review). GPP—Good Practice Points (percentile of agreement from survey, all ≥75%).

## 5 CONCLUSION

These are the first international multidisciplinary consensus‐based recommendations for the use of KDT for children with super‐refractory status epilepticus. Recommendations are based on evidence from published literature, survey consensus agreement, or the clinical expertise of the study group.

This document is intended for use by multidisciplinary teams managing children with super‐refractory status epilepticus. The recommendations aim to standardize care internationally, but do not determine a course of action and are not mandatory.

It is important to emphasize that T should be started without delay in these settings. Considering the split view amongst survey respondents regarding starting KDT prior to screening labs results being available, local multidisciplinary teams should exercise judgment for each individual case.

It must be acknowledged that there is a paucity of published research on the use of ketogenic therapies in SRSE and the quality of available evidence is predominantly low or with “critical” limitations. There are gaps, not only in well‐designed trials, but also in applicability and stakeholder integration as part of relevant intensive care guidelines and consensus statements. As a result, many of our recommendations are necessarily based on expert consensus and survey agreement rather than high‐quality publications. This represents an important limitation but reflects the best evidence available at the time of writing. Well‐designed, adequately powered clinical trials are needed to evaluate the effectiveness of KDT in this context and to determine optimal implementation strategies.

The survey methodology used to inform several recommendations also carries inherent limitations, including potential sampling and response bias. The authors acknowledge that only a small number of intensivists responded to the survey, and no pharmacists, which may have influenced the results. A broader representation of professionals beyond dietitians, and greater inclusion of respondents from non‐English speaking countries, would have strengthened the generalizability and international relevance of these recommendations. Despite these limitations, the core recommendations remain clinically relevant and grounded in expert practice, and we hope they will support dietitians, neurologists, and intensivists in the management of children and young people with super‐refractory status epilepticus.

## CONFLICT OF INTEREST STATEMENT

RB is a consultant to Nutricia North America. J. Helen Cross has acted as an investigator for studies with Jazz/GW Pharmaceuticals, Marinus, Stoke Therapeutics, Longboard/Lundbeck, UCB/Zogenix, Ultragenyx, Encoded, and Vitaflo; has been a speaker and has served on advisory boards for Biocodex, Jazz Pharmaceuticals, Nutricia, Stoke Therapeutics, and UCB (all remuneration has been paid to her department); holds an endowed chair at the University College of London Great Ormond Street Institute of Child Health; has received grants from the National Institute for Health and Care Research (NIHR), the Engineering and Physical Sciences Research Council (EPSRC), the Great Ormond Street Hospital for Children (GOSH) Charity, LifeArc, and Epilepsy Research UK; and her research is supported by the NIHR Great Ormond Street Hospital Biomedical Research Centre. CE has received honorarium from GW Pharmaceuticals/JAZZ Pharmaceuticals and from Nutricia for educational activities, all paid to the department. NES was previously supported for a research post by Vitaflo. She has received grants from Nutricia Advanced Medical Nutrition, Vitaflo, and Matthew's Friends Charity, and honoraria from Nutricia Advanced Medical Nutrition, Vitaflo, Dr. Schaer, and Proveca. CTS is a director of FabeSmith Limited (trading as KetoSuite™) and Smith Family Limited (trading as Ketogenic Diet Therapy New Zealand). She has received grants from Callaghan Innovation and The Brydie Lauder Trust and honoraria from Nutricia Limited (NZ) and Cortex Health. EvL has benefitted from research funds from Dr. Schaer and Vitaflo paid via the Ketogenic Dietitians Research Network. The remaining authors have no conflicts of interest.

## ETHICS STATEMENT

This project had no patient involvement, nor use of patient data, and so no ethics approval was required. We confirm that we have read the Journal's position on issues involved in ethical publication and affirm that this report is consistent with those guidelines.

## Supporting information


Data S1.

## Data Availability

The data that support the findings of this study are openly available in Supporting Information.
