# Supplementary material for: Ketogenic diet therapy for children with super‐refractory status epilepticus in intensive care: International clinical practice recommendations
Source: Epilepsia Open. 2026 May 14;11(4):1354–69. doi: 10.1002/epi4.70284 (PMC13394031; doi:10.1002/epi4.70284)
Supplement: Supplementary file 1 — Data S1. [file EPI4-11-1354-s001.docx]

**Supplementary Material: Survey Questions**

Q1. What is your job role?

Q2. Who is your registered professional body?

Q3. What is your clinical specialty?

Q4. Do you work with children, adults, or both?

Q5. For how many years have you managed patients in the intensive/critical care unit with status epilepticus?

Q6. The ketogenic diet should be started within one week of new onset refractory status epilepticus (NORSE) in accordance with the international consensus recommendations for management of NORSE including febrile infection-related epilepsy syndrome (FIRES) (Wickström et al., 2022).

Q7. The ketogenic diet should be started within one week of status epilepticus (SE) where intravenous anti-seizure medications (ASM) are unable to be weaned in the intensive care unit.

Q8. In the absence of risk factors for fatty acid oxidation disorders (hypoglycaemic episodes, deranged liver enzymes, elevated creatine kinase), consider initiating the diet prior to confirmation of normal results of carnitine profile and/or organic acids to prevent delays to treatment.

Q9. Where risk factors for disorders of fatty acid oxidation disorders (hypoglycaemic episodes, deranged liver enzymes, elevated creatine kinase) are present, await confirmation of normal results of carnitine profile and/or organic acids.

Q10. Prior to initiation of ketogenic diet, all carbohydrates should be removed from medications, supplements and fluids where possible.

Q11. The ketogenic diet should be introduced over a period of 12 - 48 hours

Q12. The ketogenic diet should be introduced using which of the following advancement plans:

Q13. International intensive care guidelines (ESPNIC; ASPEN &amp; SCCM; or ESPEN) should be followed with regards to route of feeding and progression of feeding (Tume et al, 2020; Mehta et al, (2017); Singer et al, 2018).(N.B.: Some centres recommend fasting and give different protocols for progression of feeds (Park, Lee and Lee, 2019))

Q14. International intensive care guidelines (ESPNIC; ASPEN; SCCM; or ESPEN) should be followed with regards to calculation of energy requirements (Tume et al, 2020; Mehta et al, 2017; Singer et al, 2018)

Q15. Protein provision should meet at least the safe low intake recommended by the Joint WHO/FAO/UNU Expert Consultation (2007). N.B. This is accepting that the gold standard would be to meet estimated requirements as per international intensive care guidelines Tume et al, 2020 (ESPNIC); Mehta et al 2017 (ASPEN; SCCM); Singer et al, 2018 (ESPEN).

Q16. Micronutrient requirements should meet at least 80% of reference nutrient intakes (RNI) from day 1 of treatment.

Q17. The ketogenic diet should be started at a 3:1 ratio in infants &lt;18 months, providing that the minimal protein requirement can be met. (Farias-Moeller et al., 2016; van der Louw et al., 2016).

Q18. The ketogenic diet should be started at a 4:1 ratio in older children &gt;18 months, providing that the minimal protein requirement can be met. (Farias-Moeller et al., 2016).N.B. Higher ratios have been reported (4.6:1 by Arayakarnkul and Chomtho, (2019); 5.0:1 by Appavu et al. (2016); 4.25:1 by Worden, Abend and Bergqvist (2020))

Q19. A higher dietary ratio than 4:1 can be considered to compensate for carbohydrates in medications (Haney et al., 2019)

Q20. The dietary prescription should be reviewed daily until stable ketosis (defined later) is achieved.

Q21. If intravenous fluids are required a carbohydrate-free, isotonic solution is advised; or isotonic dextrose at the lowest concentration of glucose needed to maintain the ketosis target and normoglycaemia (GOSH guidelines, unpublished)

Q22. Blood glucose should be monitored 4-6 hourly during diet initiation.

Q23. Serum ketone levels are the preferred method for monitoring ketones.

Q24. Serum ketones should be monitored 4-6 hourly while weaning onto the ketogenic diet

Q25. The replacement of some long chain triglyceride (LCT) fat with medium chain triglyceride (MCT) fat should be considered if ketosis is not achieved at the maximum ratio.

Q26. Monitoring of biochemistry and clinical side effects should be led by the intensive care medical team in accordance with Kossoff et al. (2018).

Q27. To reduce the risk of constipation, fluid allowance should be optimised where possible in agreement with the intensive care team

Q28. In the case of constipation, dietary fibre intake should be reviewed and optimised where possible, alongside medical management.

Q29. In the case of diarrhoea with no medical cause, a reduction in MCT fat should be considered.

Q30. In the case of vomiting or reflux, dietary adjustments such as rate and volume of feed should be reviewed.

Q31. Stable ketosis can be defined as ketones >2mmol/l for a period of 48hours.

Q32. The diet should be continued for a minimum of two weeks once stable ketosis is achieved (Schoeler et al., 2021; Worden, Abend and Bergqvist, 2020)

Q33. If efficacy is unclear at two weeks, continuing the diet for a further two weeks (to a total of four weeks) should be considered if not contraindicated (Worden, Abend and Bergqvist, 2020).

Q34. If the diet is not effective, the wean of the diet should take place over 24 - 48hours.

Q35. Please enter any additional comments.

**Supplementary Table S1: Summary of included studies from literature review, n=22**

| **Author (year)** | **Article type** | **Adults/Paediatrics** | **Main Findings** |
| --- | --- | --- | --- |
| Al-Yousif et al. (2021)  (1) | Narrative Review | n/a | Narrative review summarising studies characterizing endogenous glucose production in critical illness. Critical illness can disrupt endogenous glucose production through multiple mechanisms, including medications administered as part of clinical care. Endogenous glucose production contributes to hyperglycemia in critical illness when abnormally elevated and to hypoglycemia when abnormally depressed, each of which has been independently associated with increased mortality. |
| Appavu et al. (2016)  (2) | Retrospective cohort | Paediatrics | Nine of ten patients aged 2–16 years with SRSE achieved resolution of status epilepticus on KD. |
| Arayakarnkul et al. (2019)  (3) | Retrospective cohort | Paediatrics | 13 of 17 patients aged 1 month–13 years with SRSE were initiated on KD, as adjunctive treatment. Eight of nine cases achieved seizure cessation on KD; four cases initially had complete seizure control with medications,  but anaesthetic reduction after 24–48 h of seizure control  failed at least twice – all four cases in this group were able to  stop continuous infusion of anaesthesia after starting KD. |
| Berger et al. (2019)  (4) | Position paper | n/a | This position paper summarises theoretical and practical aspects of the monitoring of artificial nutrition and metabolism in critically ill patients, thereby completing ESPEN guidelines on intensive care unit nutrition. |
| Caraballo et al. (2014)  (5) | Retrospective cohort | Paediatrics | Ten pediatric patients were identified. Of 10 patients with refractory SE started on KD, seizures stopped in two patients and five patients had 50–75% seizure reduction within 2–5 days following the onset of ketonuria and within 5–7 days following the onset of the diet. Three patients had a <50% seizure reduction and all of them had severe adverse events so the diet was discontinued. Seven patients remained on the diet for 6 months to 3 years (mean 1.5 years). |
| Chiu et al. (2020)  (6) | Case Report | Paediatrics | A case of an 11 year old boy with small vessel cPACNS complicated by SRSE is presented. KD was tried, as well as multiple antiseizure medications, but ‘effective ketosis was difficult because of his multiple infusions’. |
| Farias-Moeller et al. (2017)  (7) | Retrospective cohort | Paediatrics | Nine children with SRSE who initiated KD in the ICU were identified. Five of these children were seizure-free one week after KD initiation. |
| Gomes et al. (2018)  (8) | Guideline | n/a | Consensus protocol on the treatment of SRSE in level III ICUs, focusing on the treatment strategies to control clinical and electroencephalographic epileptic activity. KDs were included as an ‘alternative therapeutic strategy’ recommended in cases of total absence of clinical response, with very low level of evidence. |
| Gunst et al. (2023)  (9) | Randomised controlled trial | Paediatrics | Secondary analysis of a multi-centre randomised controlled trial to determine whether hypoglycemia in PICU was associated with mortality and 4-year neurodevelopmental outcome.  Hypoglycemia univariably associated with higher mortality in PICU, at 90 days and 4 years after randomisation, but not when adjusted for risk factors. Critically ill children exposed to hypoglycemia in PICU were at higher risk of impaired executive functions after 4 years, especially in cases of spontaneous/recurrent hypoglycemia. |
| Haney et al. (2019)  (10) | Proof-of-principle prospective case-control study | Paediatrics | This proof-of-principle study demonstrated feasibility of compensating for carbohydrates in liquid medications by increases in the daily ketogenic ratio. |
| Honarmand et al. (2024)  (11) | Guideline | Adults and Paediatrics | Society of Critical Care Medicine Guidelines on Glycemic Control for Critically Ill Children and Adults. The panel achieved consensus regarding a preference for an insulin infusion for the acute management of hyperglycemia with titration guided by an explicit clinical decision support tool and frequent (≤ 1 hr) monitoring intervals during glycemic instability to minimize hypoglycemia and against targeting intensive glucose levels. |
| Joint WHO/FAO/UNU (2007)  (12) | Guideline | Adults and Paediatrics | This report is a reference for the adequacy of population food intakes; set national food and nutrition guidelines and regulations on the protein and amino acid content of industrially processed foods; determine nutrient needs, and evaluate and ensure the adequacy of rations for vulnerable groups. |
| Kossoff et al. (2018)  (13) | Guideline | Paediatrics | Updated clinical management best practice recommendations for children with epilepsy on KD. |
| Li et al. (2021)  (14) | Case Report | Paediatrics | The case is described of a previously healthy 3-year-old male with new-onset SE admitted to the ICU for 55 days. KD was initiated on day 14, and the patient achieved complete neurological recovery following KD. |
| Toro-Polo et al. (2018)  (15) | Retrospective cohort | Paediatrics | In this study of 552 ICU patients, the association was analysed between glycemia levels upon pediatric ICU admission and patient mortality. The authors concluded that there was an increased risk of death at discharge in patients who developed hypoglycemia and hyperglycemia upon admission to the ICU. Certain glucose ranges (> 200mg/dL (> 11.1mmol/L) and < 65mg/dL (3.61mmol/L)) have high specificity as predictors of death at discharge. |
| Martin‐McGill et al. (2019)  (16) | Survey | Adults and Paediatrics | A survey of KD centres across the UK and Ireland exploring the definition of MKDs. The average macronutrient profile was 75% fat and 5% carbohydrate, with protein ad libitum. Carbohydrate and fat intake targets are either weighed or determined by ‘household measures.' |
| Park et al. (2019)  (17) | Retrospective cohort | Paediatrics | Of 16 patients with SRSE treated with KD, 9 achieved seizure freedom,  six (37.5%) reported >50% seizure reduction, and one (6.2%) had <50% seizure improvement. There was no significant change in the number of antiepileptic drugs. The most commonly encountered complication during KD was gastrointestinal disturbance. The authors concluded that KD is an effective alternative therapeutic strategy for SRSE patients in ICUs with adequate efficacy and safety in reducing seizure frequency and weaning from prolonged mechanical ventilation, although functional outcome was not favorable for most patients. Close monitoring for potential adverse effects is critical. |
| Schoeler et al. (2021)  (18) | Systematic Review and case series | Paediatrics | The experience of eight children with SRSE due to FIRES, treated with KD was described. Seven of the 8 children experienced adverse side effects. SE ceased in six children after mean 25+/− 9.4 days post onset, but other treatments were often started concomitantly and all children started other treatments post-KD.  31 articles were included in the literature review, of which 24 were “medium” or “low” quality. SRSE resolved in 85/141 (60%) children after mean 6.3 days (range 0–19) post SE onset, but it is unclear whether further treatments were initiated post-KD. The authors concluded that KD should be considered as an early treatment option in this group, given that serious adverse side effects seem rare and response rates are (cautiously) favorable. |
| van der Louw et al. (2016)  (19) | Guideline | Paediatrics | Recommendations for use of KD in infants with epilepsy, based on published evidence and expert opinion. Recommendations encompass patient selection, pre-KD counseling and evaluation, specific nutritional requirements, preferred initiation, monitoring of adverse effects at initiation and follow-up, evaluation and KD discontinuation. |
| Faustino et al. (2012)  (20) | Systematic Review | Paediatrics | The literature on hypoglycemia in critically ill neonates and children was reviewed. Based on associated outcomes, the authors suggest defining hypoglycemia as <40-45 mg/dl in neonates and <60-65 mg/dl in children. Below these thresholds, hypoglycemia is associated with worse neurological outcomes, increased intensive care unit stay, and increased mortality. |
| Wickström et al. (2022)  (21) | Guideline | Adults and Paediatrics | 85 recommendation statements achieved consensus for management of NORSE, including FIRES. Recommendations are divided into: disease characteristics, diagnostic testing and sampling, acute treatment, treatment in the post-acute phase, and research, registries, and future directions. |
| Worden et al. (2020)  (22) | Retrospective cohort | Paediatrics | KD was initiated in 12 patients with SRSE, 8 patients with EE, and 9 patients with EE with SRSE. KD was initiated after a median of 9 days. Ketosis was achieved 2 days faster in fasted patients  (p<0.0001). All patients had at least one KD-related adverse effect, most often hypoglycemia, constipation, or acidosis. There was ≥50 % reduction in seizure frequency compared to prior to KD initiation by 1 week in 17/28 patients, seizure-freedom by 2 weeks in 7/28 patients, and weaned off anesthetics in 11/17 patients. All KD responders at 1 month had continued response at 6 months. |

Key:

cPACNS = Childhood primary angiitis of the central nervous system

EE = epileptic encephalopathy

FIRES = febrile infection-related epilepsy syndrome

ICU = intensive care unit

KD = ketogenic diet

MKD = modified ketogenic diet

NORSE = new onset refectory status epilepticus

SE = status epilepticus

SRSE = Super refractory status epilepticus

**References** (**Supplementary Table S1):**

1. Al-Yousif N, Rawal S, Jurczak M*, et al.* Endogenous Glucose Production in Critical Illness. *Nutr Clin Pract*. 2021, **36**(2) 344-59.

2. Appavu B, Vanatta L, Condie J*, et al.* Ketogenic diet treatment for pediatric super-refractory status epilepticus. 2016.

3. Arayakarnkul P, Chomtho K. Treatment options in pediatric super-refractory status epilepticus. *Brain Dev*. 2019, **41**(4) 359-66.

4. Berger MM, Reintam-Blaser A, Calder PC*, et al.* Monitoring nutrition in the ICU. *Clin Nutr*. 2019, **38**(2) 584-93.

5. Caraballo RH, Flesler S, Armeno M*, et al.* Ketogenic diet in pediatric patients with refractory focal status epilepticus. *Epilepsy Res*. 2014, **108**(10) 1912-6.

6. Chiu M, Datta A. Childhood Small Vessel Primary Angiitis of the Central Nervous System: A Treatable Cause of Super-refractory Status Epilepticus. *J Child Neurol*. 2020, **35**(1) 31-6.

7. Farias-Moeller R, Bartolini L, Pasupuleti A*, et al.* A Practical Approach to Ketogenic Diet in the Pediatric Intensive Care Unit for Super-Refractory Status Epilepticus. *Neurocritical care*. 2017, **26**(2) 267-72.

8. Gomes D, Pimentel J, Bentes C*, et al.* Consensus Protocol for the Treatment of Super-Refractory Status Epilepticus. *Acta Med Port*. 2018, **31**(10) 598-605.

9. Gunst J, De Bruyn A, Jacobs A*, et al.* The association of hypoglycemia with outcome of critically ill children in relation to nutritional and blood glucose control strategies. *Crit Care*. 2023, **27**(1) 251.

10. Haney CA, Charpentier A, Turner Z*, et al.* A Proof-of-Principle, Case-Control Study to Compensate for Potential Carbohydrates in Liquid Antiseizure Drugs in Children on the Ketogenic Diet. 2019.

11. Honarmand K, Sirimaturos M, Hirshberg EL*, et al.* Society of Critical Care Medicine Guidelines on Glycemic Control for Critically Ill Children and Adults 2024. *Crit Care Med*. 2024, **52**(4) e161-e81.

12. World Health Organization. Protein and amino acid requirements in human nutrition. Report of a joint WHO/FAO/UNU expert consultation. . 2007.

13. Kossoff EH, Zupec-Kania BA, Auvin S*, et al.* Optimal clinical management of children receiving dietary therapies for epilepsy: Updated recommendations of the International Ketogenic Diet Study Group. *Epilepsia Open*. 2018, **3**(2) 175-92.

14. Li WJ, Xue CL, Zhang Y*, et al.* Ketogenic diet (KD) therapy in the acute phase of febrile infection-related epilepsy syndrome (FIRES): a case report. *Transl Pediatr*. 2021, **10**(9) 2392-7.

15. Toro-Polo LM, Ortiz-Lozada R, Chang-Grozo S*, et al.* Glycemia upon admission and mortality in a pediatric intensive care unit. *Revista Brasileira de terapia intensiva*. 2018, **30**(4) 471–8.

16. Martin-McGill KJ, Lambert B, Whiteley VJ*, et al.* Understanding the core principles of a 'modified ketogenic diet': a UK and Ireland perspective. *J Hum Nutr Diet*. 2019, **32**(3) 385–90.

17. Park EG, Lee J, Lee J. The ketogenic diet for super-refractory status epilepticus patients in intensive care units. *Brain Dev*. 2019, **41**(5) 420-7.

18. Schoeler NE, Simpson Z, Zhou R*, et al.* Dietary Management of Children With Super-Refractory Status Epilepticus: A Systematic Review and Experience in a Single UK Tertiary Centre. *Front Neurol*. 2021, **12** 643105.

19. van der Louw E, van den Hurk D, Neal E*, et al.* Ketogenic diet guidelines for infants with refractory epilepsy. *Eur J Paediatr Neurol*. 2016, **20**(6) 798-809.

20. Faustino EV, Hirshberg EL, Bogue CW. Hypoglycemia in critically ill children. *J Diabetes Sci Technol*. 2012, **6**(1) 48-57.

21. Wickstrom R, Taraschenko O, Dilena R*, et al.* International consensus recommendations for management of New Onset Refractory Status Epilepticus (NORSE) incl. Febrile Infection-Related Epilepsy Syndrome (FIRES): Statements and Supporting Evidence. *Epilepsia*. 2022, **63**(11) 2840-64.

22. Worden LT, Abend NS, Bergqvist AGC. Ketogenic diet treatment of children in the intensive care unit: Safety, tolerability, and effectiveness. *Seizure*. 2020, **80** 242-8.

**Supplementary Table S2: EPHPP Quality Assessment Appraisal of Included Quantitative Studies**

| **Quantitative reference** | **A:**  **Selection Bias** | **B:**  **Study Design** | **C:**  **Confounders** | **D:**  **Blinding** | **E:**  **Data Collection Methods** | **F:**  **Withdrawals and Drop-outs** | **Global Rating** |
| --- | --- | --- | --- | --- | --- | --- | --- |
| Appavu, 2016 | Moderate | Moderate | Weak | Moderate | Weak | Moderate | Weak |
| Arayakarnkul, 2019 | Moderate | Moderate | Weak | Moderate | Weak | Moderate | Weak |
| Caraballo, 2014 | Moderate | Moderate | Weak | Moderate | Weak | Moderate | Weak |
| Chiu, 2019 | Moderate | Moderate | Weak | Moderate | Weak | Moderate | Weak |
| Farias-Moeller, 2016 | Moderate | Moderate | Weak | Moderate | Weak | Moderate | Weak |
| Gunst, 2023 | Strong | Strong | Strong | Moderate | Strong | Strong | Strong |
| Haney, 2019 | Moderate | Moderate | Weak | Moderate | Weak | Weak | Weak |
| Li, 2021 | Moderate | Weak | Weak | Moderate | Weak | Moderate | Weak |
| Toro-Polo, 2018 | Moderate | Moderate | Weak | Moderate | Weak | Moderate | Weak |
| Park, 2019 | Weak | Weak | Weak | Moderate | Weak | Moderate | Weak |
| Worden, 2020 | Moderate | Weak | Weak | Moderate | Weak | Moderate | Weak |

**Supplementary Table S3: AMSTAR II Quality Appraisal of Included Systematic Reviews**

| **Systematic review reference** | **Protocol pre-registered (CD2)** | **Adequate search (CD4)** | **Justified exclusions (CD7)** | **RoB assessment (CD9)** | **Bias considered in interpretation (CD13)** | **Overall AMSTAR-2 rating** |
| --- | --- | --- | --- | --- | --- | --- |
| Al-Yousif, 2021 | Critical flaw | Critical flaw | Critical flaw | Critical flaw | Critical flaw | Critically low |
| Schoeler, 2021 | Critical flaw | Non-critical weakness | Critical flaw | Critical flaw | Critical flaw | Critically low |
| Vincent, 2012 | Critical flaw | Non-critical weakness | Critical flaw | Critical flaw | Critical flaw | Critically low |

**Supplementary Table S4: AGREE II Domain Scores for Included Clinical Guidelines**

| **Guideline reference** | **D1 Scope & Purpose** | **D2 Stakeholder** | **D3 Rigor** | **D4 Clarity** | **D5 Applicability** | **D6 Editorial Independence** |
| --- | --- | --- | --- | --- | --- | --- |
| Berger, 2019 | 80.6 | 41.7 | 21.8 | 66.7 | 29.2 | 70.8 |
| Gomes, 2018 | 88.9 | 22.2 | 35.5 | 72.2 | 20.8 | 100.0 |
| Hon, 2024 | 97.2 | 88.9 | 91.7 | 889 | 78.6 | 87.5 |
| Kossoff, 2018 | 72.2 | 58.3 | 60.4 | 77.8 | 45.8 | 100.0 |
| Van der Louw, 2016 | 100.0 | 86.1 | 69.8 | 97.2 | 77.1 | 91.7 |
| Wickstrom, 2022 | 100.0 | 86.1 | 82.3 | 100.0 | 79.2 | 95.8 |

**Supplementary Table S5: Commonly-used prescribable products available for support of ketogenic diets (in alphabetical order within each section)**

| **Product** | **Company** | **Description** | **Indications*** |
| --- | --- | --- | --- |
| **Nutritionally complete** | | | |
| Ketobiota 2.5:1 | Dr Schaer/Kanso | Powdered, texture can be adapted (e.g. liquid or yogurt) with 60% MCT. Enriched with 11 vitamins | Suitable from 3 years of age onwards |
| K.Flo 4:1 | Nestle Health Science/Vitaflo | Ready to use fibre‐enriched liquid feed available in vanilla | Suitable from 3 years of age onwards |
| Ketocal 2.5:1 LQ | Nutricia | Ready to use fibre‐enriched liquid feed available in vanilla | Suitable as a sole source of nutrition in children aged 8 years to adults or as a supplement |
| Ketocal 4:1 LQ | Nutricia | Ready to use fibre enriched liquid feed available in vanilla and unflavoured varieties | Suitable as a sole source of nutrition in children aged 1–10 years or as a supplement for those over 10 years and adults |
| Ketocal 3:1 powder | Nutricia | Powdered feed, fibre‐free, enriched with LCPs, unflavoured. The standard feed concentration is 9.5% | Suitable as a sole source of nutrition in infants from birth to 6 years or as a supplement in those over 6 years (UK version). |
| Ketocal 4:1 powder | Nutricia | Powdered feed enriched with fibre and LCPs available in vanilla and unflavoured varieties. The standard feed concentration is 14.3% | Suitable as a sole source of nutrition, or as a supplementary feed, in children over 1 year. For sip and tube feeding |
| KetoEpi 2:1 | Dr Schaer/Kanso | Ready to use liquid formula with 65% MCT, allergen-free | Suitable from 3 years of age onwards Suitable as a sole source of nutrition |
| Ketonia | Namyang Dairy Products Co., Ltd. | Ready to use liquid formula for oral or enteral use in infants and young children | Suitable from birth |
| Ketovie 3:1 | Cortex Health / Ajinomoto Cambrooke Inc | Ready to use partially hydrolyzed whey protein with 20% MCT.  Enriched with prebiotic fibre and carnitine. | Suitable as a sole source of nutrition in children from 1 year of age |
| Ketovie 4:1 | Cortex Health / Ajinomoto Cambrooke Inc | Ready to use fibre, carnitine and citrate enriched liquid feed with 25% MCT. Available in unflavoured, vanilla or chocolate flavour | Suitable as a sole source of nutrition in children from 1 year of age |
| Ketovie Peptide 4:1 | Cortex Health / Ajinomoto Cambrooke Inc | Extensively hydrolyzed whey protein.  Ready to use fibre, carnitine and citrate enriched liquid feed with 15% MCT. | Suitable as a sole source of nutrition in children from 1 year of age |
| Ketovie 4:1 Plant-Based Protein | Cortex Health / Ajinomoto Cambrooke Inc | Pea protein  Ready to use fibre, carnitine and citrate enriched liquid feed with 25% MCT. | Milk and soy allergies.  Suitable as a sole source of nutrition in children from 1 year of age |
| K.Yo | Nestle Health Science/Vitaflo | Ready to eat semi‐solid food | Suitable from 3 years of age onwards. Suitable as a sole source of nutrition up to 10 years of age |
| **Carbohydrate free formula** | | | |
| Carb free mix | Nutricia | Powdered feed very low in carbohydrate | Suitable for infants and children |
| RCF | Abbott | Liquid feed very low in carbohydrate, soy protein | Milk allergy  Suitable from birth |
| **Fat modules** | | | |
| Calogen | Nutricia | 50% LCT fat emulsion | Suitable from birth |
| K.Quik | Nestle Health Science/Vitaflo | Ready to use 20% emulsion of MCT | Suitable from 3 years of age |
| Liquigen | Nutricia | 50% MCT emulsion | Suitable from birth |
| MCT oil | Nutricia | Liquid containing only a mixture of MCT | Suitable from birth |
| MCT oil (77% and 100%) | Dr Schaer/Kanso | Liquid containing only a mixture of MCT | Suitable from birth MCT 100% must be used raw |
| **Protein modules** | | | |
| Beneprotein | Nestle Health Science | Powdered milk based high protein supplement | Suitable from birth |
| Complete Amino Acid Mix | Nutricia | Powdered mix of essential and non-essential amino acids | Suitable from birth |
| MCT Procal | Nestle Health Science/Vitaflo | Neutral tasting protein  powder supplement high  in MCT | Suitable from 3 years of age |
| ProSource TF | Nutrinovo | Liquid high protein milk‐free (beef collagen derivative) supplement for tube feeding | Suitable from 3 years of age |
| Protifar | Nutricia | Powdered milk based high protein supplement | Suitable from birth |
| **Carbohydrate modules** | | | |
| Polycal/Polyjoule | Nutricia | Powdered unflavoured carbohydrate supplement | Suitable from 1 year of age |
| Super Soluble Maxijul | Nutricia | Powdered neutral flavoured carbohydrate energy source | Suitable from birth |
| Vitajoule | Nestle Health Science/Vitaflo | Powdered unflavoured carbohydrate supplement | Suitable from birth |
| **Other** | | | |
| DeliMCT creams (champignons, tomatoes, classical) | Dr Schaer/Kanso | Ready to use, enriched with MCT (ranging from 85% to 95%) | Suitable from 3 years of age |
| DeliMCT Cacaobar | Dr Schaer/Kanso | Ready to eat, with 33% MCT, enriched in fiber. Ketogenic ratio 5.2:1 | Suitable from 3 years of age |
| KetoClassic 3:1 Bisk | Ketocare foods | 3:1 ratio high fat, high fiber food | Suitable from 3 years of age |
| KetoClassic 3:1 breakfast Porridge | Ketocare foods | 3:1 ratio, high fat, high fiber, ready prepared meal | Suitable from 3 years of age |
| KetoClassic 3:1 breakfast Muesli | Ketocare foods | 3:1 ratio, high fat, high fiber, ready prepared meal | Suitable from 3 years of age |
| KetoClassic 3:1 meal Savoury | Ketocare foods | 3:1 ratio, high fat, high fiber solid meal | Suitable from 3 years of age |
| KetoClassic 3:1 meal Chicken | Ketocare foods | 3:1 ratio, high fat, ready prepared meal | Suitable from 3 years of age |
| KetoClassic 3:1 meal Bolognese | Ketocare foods | 3:1 ratio, high fat, ready prepared meal | Suitable from 3 years of age |
| Keto Peptide | Functional Formularies | Whole foods-based formula (2.43:1 ratio). Includes peptide proteins. | No specific indications given |
| KetVit | Dr Schaer/Kanso | Ready to eat creamy food, with 44% MCT. Enriched with minerals, vitamins and fiber  Ketogenic ratio 5.7:1 | Suitable from 3 years of age.  Not suitable as a sole source of nutrition. |
| MCTfiber | Dr Schaer/Kanso | Powder, 60% MCT and ketogenic ratio 7.2:1. Added with soluble fiber | Suitable from 3 years of age |
| MCT Margarine 83% | Dr Schaer/Kanso | Ready to eat, with 83% MCT. Enriched with omega-3 + omega-6, vitamins A, D, E, folate, vitamin B12 | Suitable from 1 years of age  Maximum temperature 180° |

LCP, long chain polyunsaturated fatty acids; MCT, medium chain triglycerides; LCT, long chain triglycerides

*Indications may vary between countries

**Supplementary Table S6: Example initiation of ketogenic diet in pediatric intensive care unit**

| **Patient details:** 4-year-old child, with a history of Leigh’s disease, gastrostomy in situ to support nutritional and fluid requirements. Admission to pediatric intensive care unit in super-refractory status epilepticus.  Normal intake 700ml of 1.2kcal pediatric formula providing 819kcal, 25.5g protein and 1400ml fluid daily plus some oral diet providing approximately 100kcal daily. History of reflux and constipation and recent transition to formula including blended real food which has been helpful.  Weight = 18kg on the 9^th^ centile |
| --- |
| **Target classical ketogenic diet prescription: 3:1 ratio**  Estimated requirements whilst ventilated and sedated: to aim for 819kcal and 19.7g protein (WHO/FAO/UNU 1985), 980 ml fluid (70% of normal fluid requirements due to being intubated and ventilated)  **Goal macronutrients: 78g fat, 20g protein and 6g carbohydrate** |

**Day 1**

Parents anxious about adjustment to feeds due to symptoms of reflux, discomfort and constipation previously. Agreed to combine formulas for a gradual transition.

|  | Fat (g) | Protein (g) | Carbohydrate (g) |
| --- | --- | --- | --- |
| 1.5:1 classical ratio (Target: 70g fat, 20g protein, 27g carbohydrate) | | | |
| 415ml 1.5kcal/ml 4:1 ketogenic formula | 61 | 14.1 | 1.4 |
| 180ml 1.2kcal/ml standard enteral formula | 9 | 6.5 | 25.3 |
| 105ml Water |  |  |  |
| **Total** | 70 | 20.6 | 26.7 |
| To feed continuously 30 ml/hr to provide 700 ml in 24 hours  Additional fluids to be provided by medications and IV fluids, avoiding glucose | | | |

**Day 2**

|  | Fat (g) | Protein (g) | Carbohydrate (g) |
| --- | --- | --- | --- |
| 2.25:1 classical ratio (Target: 78g fat, 20g protein, 14.5g carbohydrate) | | | |
| 500ml 1.5kcal/ml 4:1 ketogenic formula | 73.5 | 17 | 1.75 |
| 90ml 1.2kcal/ml standard enteral formula | 4.5 | 3.24 | 12.6 |
| 110ml Water |  |  |  |
| **Total** | 78 | 20.24 | 14.35 |
| To feed continuously 30 ml/hr to provide 700 ml in 24 hours  Additional fluids to be provided by medications and IV fluids, avoiding glucose | | | |

**Day 3**

|  | Fat (g) | Protein (g) | Carbohydrate (g) |
| --- | --- | --- | --- |
| 3:1 classical ratio (Target: 78g fat, 20g protein, 6g carbohydrate) | | | |
| 530 ml 1.5kcal/ml 4:1 ketogenic formula | 77.9 | 18 | 1.85 |
| 4g carbohydrate powder | - | - | 3.8 |
| 2.5g protein powder | - | 2.2 | - |
| 170ml of water | - | - | - |
| **Total** | 77.9 | 20.2 | 5.72 |
| To feed continuously 30 ml/hr to provide 700 ml in 24 hours, aiming to move to pump assisted boluses as tolerated e.g. 60ml every 2 hours  Additional fluids to be provided by medications and IV fluids, avoiding glucose | | | |

**Supplementary Table S7: Example initiation of ketogenic diet in pediatric intensive care unit**

| **Patient details:** 3-month-old infant, with a history of GRIN2D-Related Developmental and Epileptic Encephalopathy, naso-gastric tube in situ and tolerating standard infant formula. Admission to pediatric intensive care unit in super-refractory status epilepticus.  Normal intake at home around 150ml/kg/day of formula but some difficulties in feeding and reflux noted. Gaining weight but on the 0.4^th^ centile for weight, length and head circumference.  Weight = 4.7kg |
| --- |
| **Target classical ketogenic diet prescription: 3:1 ratio**  Estimated requirements whilst ventilated and sedated: to aim for 451kcal (Schofield), and 6.4-7.8g protein minimum as safe intake (WHO/FAO/UNU 1985), 494 ml fluid (70% of normal fluid requirements due to being intubated and ventilated)    **Goal macronutrients: 43.6g fat, 7.8g protein and 6.7g carbohydrate** |

**Day 1**

|  | Fat (g) | Protein (g) | Carbohydrate (g) |
| --- | --- | --- | --- |
| 2:1 classical ratio (Target: 41g fat, 7.8g protein, 12.7g carbohydrate) | | | |
| 55g 3:1 ketogenic infant formula | 37.7 | 8.5 | 4 |
| 110ml standard infant formula | 3.7 | 1.4 | 8 |
| Water to make up 500ml (90kcal/ml) |  |  |  |
| **Total** | 41.4 | 9.9 | 12 |
| To feed continuously 21 ml/hr to provide 494 ml in 24 hours | | | |

**Day 2**

|  | Fat (g) | Protein (g) | Carbohydrate (g) |
| --- | --- | --- | --- |
| 3:1 classical ratio (Target: 43.6g fat, 7.8g protein, 6.7g carbohydrate) | | | |
| 64g 3:1 ketogenic infant formula | 43.9 | 9.9 | 4.6 |
| Water to make up to 500ml |  |  |  |
| **Total** | 43.9 | 9.9 | 4.6 |
| To feed continuously 21 ml/hr to provide 494 ml in 24 hours  Decision made to allow lower carbohydrate intake as also receiving regular liquid paracetamol which contains sugar alcohols (unable to use tablet formulation due to sodium content) | | | |

**Day 3**

Not achieving ketosis, likely secondary to sugar alcohols in medication formulations. Agreed to replace part of the LCT fats with MCT to encourage ketosis

|  | Fat (g) | MCT (g) | Protein (g) | Carbohydrate (g) |
| --- | --- | --- | --- | --- |
| 3:1 classical ratio with 10% MCT (Target: 38.6g fat, 5.4g MCT, 7.8g protein, 6.7g carbohydrate) | | | | |
| 55g 3:1 ketogenic infant formula | 37.7 | - | 8.5 | 4 |
| 19ml standard infant formula | 0.6 | - | 0.25 | 1.4 |
| 10ml 50% MCT emulsion | - | 5 | - | - |
| Water to make up to 500ml | - |  | - | - |
| **Total** | 38.3 | 5 | 8.75 | 5.4 |
| To feed continuously 21 ml/hr to provide 494 ml in 24 hours | | | | |

Tolerated introduction of MCT and ketones within target range
